# Understanding the Uncanny: Both Atypical Features and Category Ambiguity Provoke Aversion toward Humanlike Robots

**DOI:** 10.3389/fpsyg.2017.01366

**Published:** 2017-08-30

**Authors:** Megan K. Strait, Victoria A. Floerke, Wendy Ju, Keith Maddox, Jessica D. Remedios, Malte F. Jung, Heather L. Urry

**Affiliations:** ^1^Social Systems Laboratory, Computer Science, University of Texas Rio Grande Valley Edinburg, TX, United States; ^2^Emotion, Brain, and Behavior Laboratory, Psychology, Tufts University Medford, MA, United States; ^3^Center for Design Research, Mechanical Engineering, Stanford University Stanford, CA, United States; ^4^Social Cognition Laboratory, Psychology, Tufts University Medford, MA, United States; ^5^Social Identity and Stigma Laboratory, Psychology, Tufts University Medford, MA, United States; ^6^Robots in Groups Laboratory, Information Science, Cornell University Ithaca, NY, United States

**Keywords:** anthropomorphism, emotion regulation, humanoid robots, human-robot interaction, uncanny valley, social robotics

## Abstract

Robots intended for social contexts are often designed with explicit humanlike attributes in order to facilitate their reception by (and communication with) people. However, observation of an “uncanny valley”—a phenomenon in which highly humanlike entities provoke *aversion* in human observers—has lead some to caution against this practice. Both of these contrasting perspectives on the anthropomorphic design of social robots find some support in empirical investigations to date. Yet, owing to outstanding empirical limitations and theoretical disputes, the uncanny valley and its implications for human-robot interaction remains poorly understood. We thus explored the relationship between *human similarity* and people's aversion toward humanlike robots via manipulation of the agents' appearances. To that end, we employed a picture-viewing task (*N*_*agents*_ = 60) to conduct an experimental test (*N*_*participants*_ = 72) of the uncanny valley's existence and the visual features that cause certain humanlike robots to be unnerving. Across the levels of human similarity, we further manipulated agent appearance on two dimensions, *typicality* (prototypic, atypical, and ambiguous) and *agent identity* (robot, person), and measured participants' aversion using both subjective and behavioral indices. Our findings were as follows: (1) Further substantiating its existence, the data show a clear and consistent uncanny valley in the current design space of humanoid robots. (2) Both category ambiguity, and more so, atypicalities provoke aversive responding, thus shedding light on the visual factors that drive people's discomfort. (3) Use of the Negative Attitudes toward Robots Scale did not reveal any significant relationships between people's pre-existing attitudes toward humanlike robots and their aversive responding—suggesting positive exposure and/or additional experience with robots is unlikely to affect the occurrence of an uncanny valley effect in humanoid robotics. This work furthers our understanding of both the uncanny valley, as well as the visual factors that contribute to an agent's uncanniness.

## 1. Introduction

By capitalizing on traits that are familiar and intuitive to people, robots designed with greater *human similarity*—both physically and behaviorally—can offer more natural and effective human-robot interactions (Duffy, 2003; Złotowski et al., 2015). For example, incorporating humanlike cues into a robot's design elicits feelings of empathy toward it (Riek et al., 2009) and causes attribution of greater agency (Gray and Wegner, 2012; Broadbent et al., 2013; Stafford et al., 2014a). In turn, this has significant prosocial outcomes such as increases in people's comfort around a robot (Sauppé and Mutlu, 2015) and their willingness to collaborate with it (Andrist et al., 2015).

With the emergence of increasingly humanlike robots, however, researchers have observed an unintended consequence: the *uncanny valley* effect (Mori et al., 2012). The valley effect, originally described by Masahiro Mori nearly a half-century ago, refers to the phenomenon wherein highly humanlike (but not prototypically human) entities provoke aversion in people (for a review, see Kätsyri et al., 2015). For example, highly humanlike robots are rated more negatively (MacDorman, 2006), avoided more frequently (Strait et al., 2015), and attributed less trustworthiness (Mathur and Reichling, 2016) than their less humanlike counterparts and humans. Moreover, such effects do not appear to be limited to adults, as valley-like effects have been observed in infants (Lewkowicz and Ghazanfar, 2012; Matsuda et al., 2012), children (Yamamoto et al., 2009), and even other primates (Steckenfinger and Ghazanfar, 2009), suggesting the general phenomenon is relatively pervasive.

Yet, the uncanny valley continues to be a poorly understood and even contentious topic in human-robot interaction (HRI) research, due to gaps in the current literature and various empirical inconsistencies. These issues stem, at least in part, from challenges inherent to conducting empirical HRI studies (in particular, the limited accessibility of robotic platforms that only partially represent the large design space). This has lead researchers to turn to more accessible alternatives, such as the use of computer-generated stimuli to make inferences about embodied counterparts (e.g., Inkpen and Sedlins, 2011) and careful case studies of only one or a few robotic platforms (e.g., Bartneck et al., 2009; Kupferberg et al., 2011; Saygin et al., 2012; Strait et al., 2014). But the small range of methodologies for investigating the valley, in turn, has lead to conflicting findings. For example, amongst studies utilizing few robots or non-embodied robot stimuli, there are both many studies which fail to find a valley effect (or find the opposite – more positive responding to the most humanlike stimuli; e.g., Bartneck et al., 2009; Kupferberg et al., 2011; Piwek et al., 2014) as well as many that confirm its existence (e.g., Saygin et al., 2012; Koschate et al., 2016; Strait et al., 2015).

Considering that the theoretical comparisons are being made across such dissimilar methodologies, it is unsurprising that inconsistencies have arisen and that gaps in the literature remain. Researchers have begun to address such shortcomings through systematic review of the literature (Kätsyri et al., 2015; Rosenthal-von der Pütten and Krämer, 2015; MacDorman and Chattopadhyay, 2016) and development of alternative methodologies. For example, two recent studies utilized picture-based stimuli (photographs depicting embodied robots) to evaluate a large portion^1^ of the current design space in humanoid robotics (Strait et al., 2015; Mathur and Reichling, 2016). In combination, recent work paints a more consistent picture in which there exists a robust uncanny valley as a function of human similarity.

Despite perspectives on the valley's existence trending toward agreement, many critical questions remain. In particular, *when, why, and how do robots fall into the uncanny valley*? Researchers have long pointed to *human similarity* as the cause of the valley effect—wherein a robot with “too much” similarity is unnerving. However, several studies indicate that similarity alone is not sufficient to cause a humanoid robot to fall into the valley. For example, Rosenthal-von der Pütten and Krämer (2014, 2015) have repeatedly shown that people respond negatively toward some instances of highly humanlike robots but positively toward others. Moreover, an experiment by Schein and Gray (2015) showed that humans too can be perceived as unnerving, suggesting that humanness (and a biologically-human appearance) is not enough to avoid the valley.

Finding the answers to these questions has particular relevance to human-robot interaction and the design of social robots. Despite the superficial nature of a robot's appearance, its appearance nevertheless substantially impacts how people perceive it and whether they are willing to interact with it (e.g., Strait et al., 2015; Mathur and Reichling, 2016). Thus, to achieve effective robot designs (or, at least, avoid ineffective ones), it remains crucial to gain better understanding of the uncanny valley and the variables (both visual and behavioral) that drive it.

### 1.1. Present work

Here, we aimed to further examine the uncanny valley as it pertains to human-robot interaction. Our contributions are three-fold: in addition to providing another experimental test of the valley's existence, we investigated what design factors cause a robot to fall into the valley. In particular, we tested two theoretically-motivated factors – *atypicality* and *category ambiguity* – for their effects on perceptions of uncanniness and people's corresponding aversion. Finally, we aimed to address an outstanding shortcoming of the current literature, namely whether people's aversion can be explained by pre-existing negative attitudes toward robots.

Recent reviews of valley literature have pointed to two explanatory mechanisms underlying the effect: *atypicality* and *category ambiguity* (cf. Kätsyri et al., 2015; MacDorman and Chattopadhyay, 2016). *Atypicality* (also called “feature atypicality” and “realism inconsistency”) refers to the presence of features unusual for an agent's category. For example, Albert Hubo is an *atypical* robot with its prototypically mechanical body combined with an atypical (highly humanlike) head. Derived from theories of perceptual mismatch, *atypicality* is proposed to underlie uncanniness via violation of expectations about how an agent should look/behave based on its category membership (Groom et al., 2009; Saygin et al., 2012). Perceptual mismatch theories thus predict that *any* atypical agent (robot or human) will provoke aversion.

*Category ambiguity*, on the other hand, refers to a difficulty in determining the category to which an entity belongs (e.g., Burleigh et al., 2013; Yamada et al., 2013). For example, people have difficulty perceiving the Geminoid HI as being a robot because of its very humanlike design (Rosenthal-von der Pütten et al., 2014). Derived from theories of categorical perception, *category ambiguity* is proposed to underlie uncanniness via doubt about what an entity is (Jentsch, 1997). Contrary to the above, categorical perception theories predict that the valley effect is greatest at category boundaries (e.g., the robot-human boundary), with aversion decreasing outwards with increasing distance.

In the present study, we observed people's subjective and behavioral aversion toward 60 distinct robots and humans using the popular picture-viewing methodology used in emotion research (see Vujovic et al., 2013), as adapted for HRI research involving social signals (Strait et al., 2015; see **Figure 2**). Participants were presented with the 60 photographs sequentially and for 12 s each. For each viewing, participants had the option to press a button if they wished to terminate the encounter early (thereby engaging in behavioral avoidance). In total, we collected participants' subjective ratings of the agents' eeriness, the frequency at which they terminated encounters with the various agents, and their reasons for terminating.

Per Mori's uncanny valley theory, we hypothesized that people would be averse to *highly humanlike* – but not prototypic – agents (**H1: Valley Hypothesis**). Specifically, relative to people of prototypically human appearances and robots of low human similarity, we expected that the appearance of highly humanlike agents would be so discomforting (as evidenced by higher ratings of eeriness; H1a) that people would avoid their encounters more frequently (H1b), and that they would report doing so due to being unnerved (H1c).

In confirming the existence of a valley in the design space included, we looked at the governing mechanisms underlying uncanniness (when, why, and how an agent falls into the valley) with two further predictions following from the literature. Specifically, we hypothesized that people would be more averse to *atypical* agents than prototypic agents (**M1: Feature Atypicality**). We also hypothesized that people would be more averse to *ambiguous* agents than prototypic agents (**M2: Category Ambiguity**). In addition to the above predictions, we explored how the two proposed mechanisms – *atypicality* vs. *ambiguity*—interact with the agents' actual category membership (whether the agent in question is a robot or a person) in provoking aversion, and further, whether people's aversive responding can be explained by pre-existing negative attitudes toward robots.

## 2. Materials and methods

Based on Mori's valley hypothesis, we expected that highly humanlike (but not prototypic) agents may be so eerie (H1a) that people avoid their encounters because due to being unnerved (H1b–c). We further predicted, based on perceptually-oriented theories of categorization and processing, that salient atypicalities (M1) and/or high category ambiguity (M2) might underlie such discomfort.

### 2.1. Design

To test our predictions, we conducted a within-subjects experiment in which we presented participants with 60 distinct agents which spanned two ontological **categories** (*robot, person*) and were of appearances that varied semi-hierarchically across two overlapping dimensions – **human similarity** (three levels: *low, high*, and *prototypic*) and **typicality** (three levels: *prototypic, atypical*, and *ambiguous*)^2^. In total, the study involved six agent conditions (with 10 agents per condition):

- 10 agents of *low* human similarity (i.e., prototypic robots such as the mechanomorphic REEM-C);
- 40 agents of *high* (but not prototypically human) human similarity:
  - 10 robots with *atypical* features (e.g., Albert Hubo),
  - 10 robots of *ambiguous* category membership (e.g., the Geminoid DK),
  - 10 people with *atypical* features (e.g., persons with bionic prostheses), and
  - 10 people of *ambiguous* category membership (persons wearing black, full-sclera contacts);
- 10 agents of *prototypic* human similarity (i.e., people of typical appearances).

Table 1 shows exemplars of each agent condition, as well as the semi-hierarchical mapping between the three manipulations (the agent's approximate human similarity and their typicality relative to their respective category membership).

**Table 1:** Exemplars of the six agent conditions, with: the agent's **category** membership reflected across the dotted y-axis (top row: *robot*; bottom: *person*); the **human similarity** manipulation shown along the x-axis (increasing left to right: from *low* similarity to *high*—inclusive of atypic and ambiguous typicality levels – to *prototypically human*), and the corresponding **typicality** levels indicated via color-coding (gray: *prototypic* for a given ontology; orange: *atypical*; and blue: *ambiguous*). **Robots** (top; from left to right): a prototypic robot (PAL ROBOTICS' REEM-C); a robot with a salient atypicality (KAIST's Albert Hubo); and a robot of ambiguous ontology (the Geminoid DK; shown, for comparison, in front of Henrik Scharfe – the person after which it was modeled). **People**: a person of prototypically human human similarity; a person with a prosthetic arm; and a person of “*ambiguous*” humanness (a person wearing black sclera contacts; face enlarged for emphasis).

**low (prototypic robot)**	**high (inclusive of both atypical and ambiguous agents)**		**prototypic (prototypic person)**
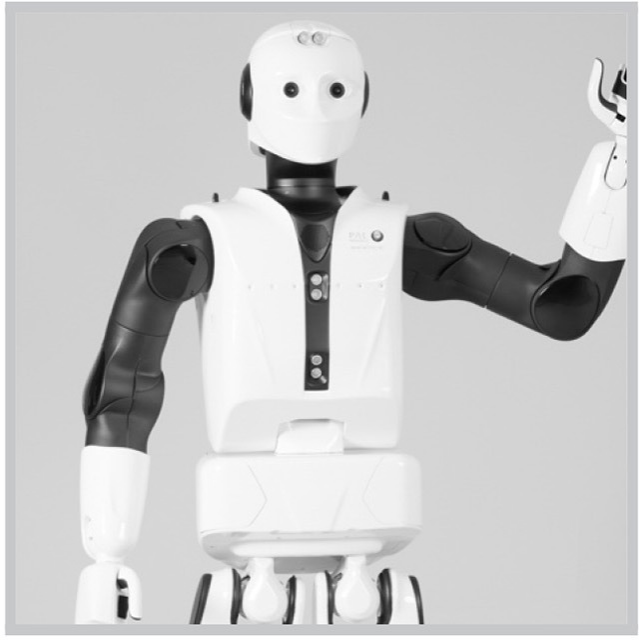	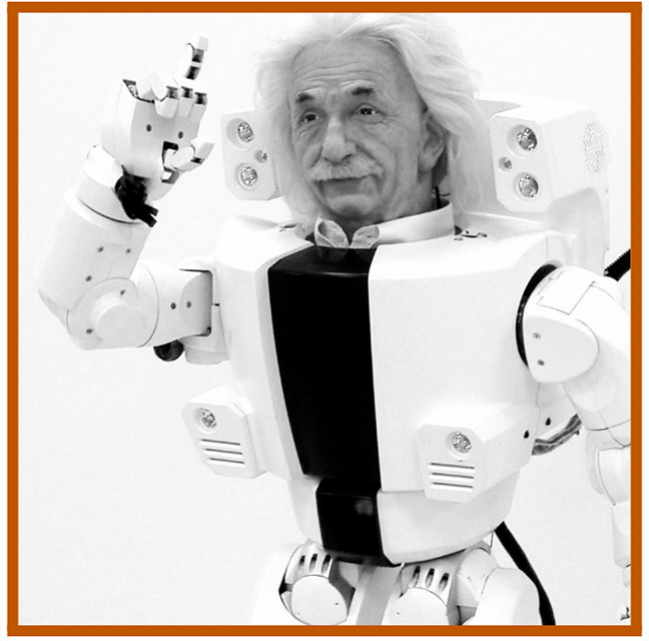	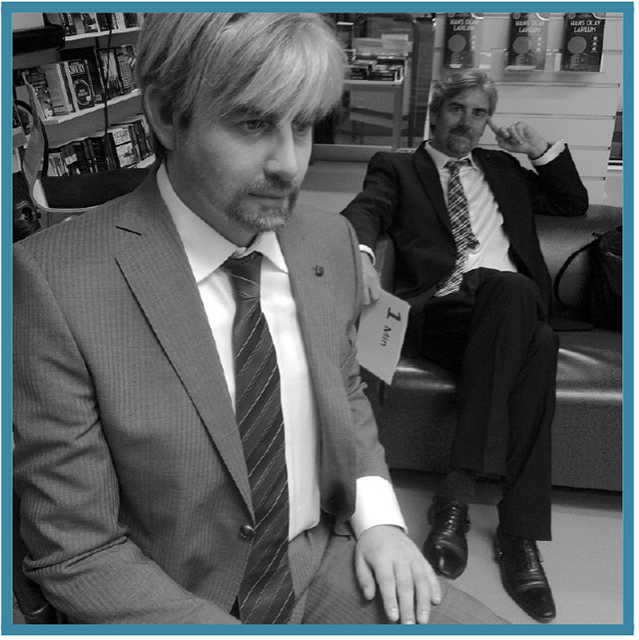	
	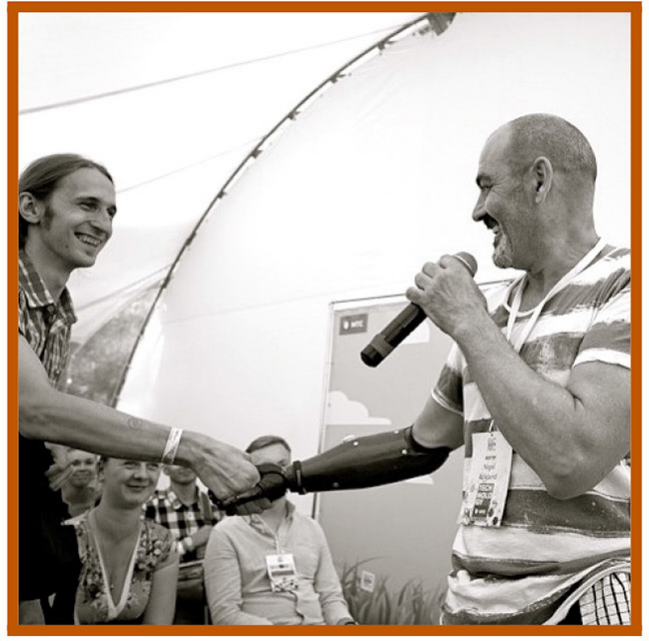	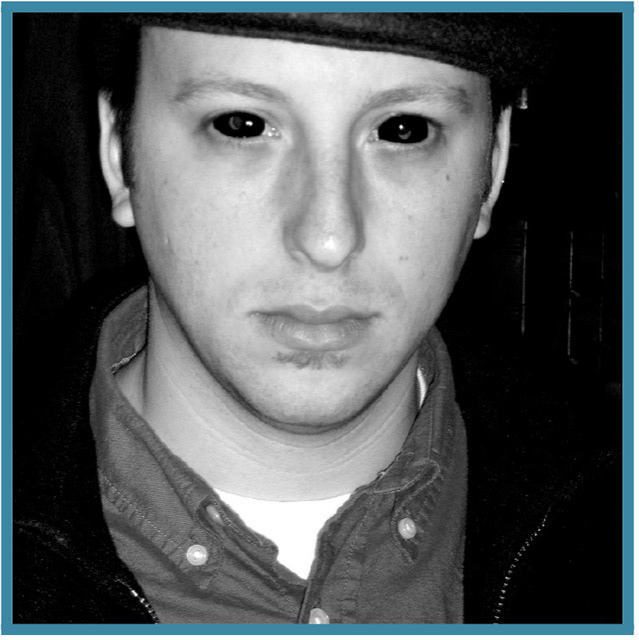	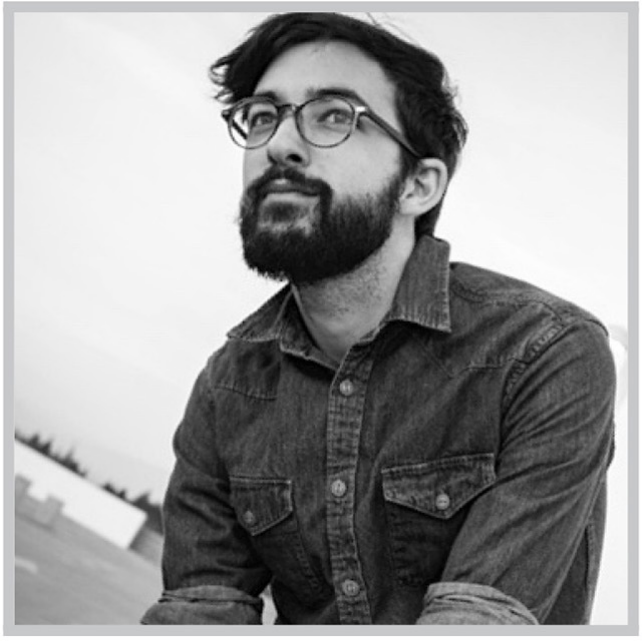

*Attribution (from top left to bottom right): shown are adaptations of photographs by JosepPAL, Dayofid, and Eirik Newth; SalganikEA and Matthew Batchelder. Original photos (https://goo.gl/38yUr1, https://goo.gl/00o07k, https://goo.gl/T7Ym4O; https://goo.gl/YfndfO, https://goo.gl/UB62Ac) available under Creative Commons Attribution-Share Alike 3.0 Unported, Attribution 2.5 Generic, or Attribution-NonCommercial-ShareAlike 2.0 Generic licenses^3^*.

#### 2.1.1. Valley hypothesis

The manipulation of the agents' human similarity was used to test whether or not there exists an uncanny valley within the current design space of humanoids and range of human appearances (H1). Note that, in testing the valley hypothesis, we collapse across the four sets of robots and people of *atypic* and *ambiguous* designations as their normalized ratings of human similarity constitute *high*—but not prototypic—human similarity. That is, they are rated as significantly more humanlike than mechanomorphic humanoids and significantly less humanlike than people of prototypic appearances.

#### 2.1.2. Mechanisms

Via the typicality manipulation, we further tested whether two mechanisms (M1: feature atypicality; M2: category ambiguity) drive the valley's effects by drilling down within the set of highly humanlike agents. Specifically, via the explicit inclusion and clustering of highly humanlike agents by those with appearances atypic for their respective category and those of ambiguous category membership, we contrasted the role of each of the two mechanisms (against prototypicality) in eliciting discomfort. Here, we additionally included the manipulation of ontological category (robot vs. person), as both the feature atypicality and category ambiguity hypotheses require that the valley effect be evident regardless of the agent's actual category membership. Thus, in testing the two hypothesis, the three typicality levels (*prototypic, atypical*, and *ambiguous*) are robot-human inclusive (e.g., *prototypic* included mechanomorphic humanoids and people of prototypically human appearances).

### 2.2. Materials

To construct a final set of high quality and relatively comparable photos, the stimuli used in this experiment were selected from an initial superset of 120 photos. The 120 photos were obtained from various academic and online sources based on strict inclusion/exclusion criteria and pretested for their fit within the six intended agent categories to reduce within-category variability.

#### 2.2.1. Set construction

We constructed our initial stimulus set via a systematic search using stringent inclusion criteria based on that developed by Mathur and Reichling (2016). The purpose of the criteria was to reduce any researcher bias that may be present in image selection (e.g., agent expression, pose, etc.). The criteria were as follows:

- Visibility: the agent's face/torso and eyes are fully visible (shown from top of head to waist; face is shown in frontal to 3/4 aspect).
- Embodiment: the agent is capable of interacting socially with humans (e.g., if a robot, the agent has been built and is capable of physical movement).
- Affect: the agent is expressionless/affect-neutral.
- Familiarity: the agent is not a replica of a well-known character or a famous person (e.g., Albert Hubo).
- Image characteristics: the resolution of the image is sufficient to yield a final cropped image of 6x6” with a resolution of 100 DPI.

We performed ten Google image searches on a single day using the following search phrases: ”humanoid robot,” ”humanlike robot,” “robot with humanlike face,” “android robot,” “highly humanlike robot,” “robot that looks human”; “black sclera contacts,” “people wearing sclera contacts,” “people with bionic prostheses,” “person candid photograph.” In collating a set of 20 *atypical* humanoids, we intentionally searched for humanoid robots with a salient mismatch in the realism of their head/torso due to greater availability of robots with this particular design. As the closest human analog (in appearance) to the set of atypical humanoids (robots with features that are atypical in terms of frequency of appearance within the humanoid design space), we specifically searched for people with a bionic prosthetic. To collate an analogous set of 20 people of “ambiguous” ontology (i.e., questionable membership in the *person* category), we intentionally searched for people wearing black, full-sclera contacts as it is a visual modification often used in media to convey different category membership (see for example: the Supernatural TV series, 2005–) and prior literature suggests that people perceive such stimuli as uncanny (Schein and Gray, 2015).

When a search returned multiple images of a particular agent, we included only the first image encountered. For each of the intended agent categories, we included the first 20 photographs satisfying inclusion criteria and depicting distinct agents. However, we note that our resulting set of *ambiguous* robots was comprised of robots that were predominately female (15 of 20) and Asian (13 of 20) in appearance.^4^ For comparability between conditions, we thus adjusted the composition of our human stimuli to reflect similar demographics. Specifically, we manually searched for replacements (per the above criteria) for the initially-selected images to adjust the gender and racial composition of the three sets of human stimuli.

#### 2.2.2. Pretesting

**Figure 1:**
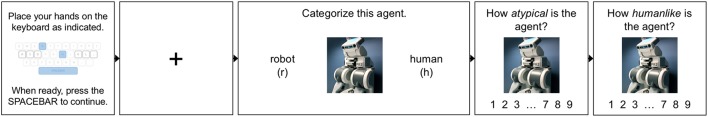
Structure of Pretesting Trials/Manipulation Checks. Each trial began with a prompt to the participant to place their hands on the keyboard as shown (with the left and right index fingers on the “r” and “h” keys respectively). When the participant was ready to continue, they pressed the spacebar to start the categorization task. After a response was entered for the categorization, participants completed two prompts for explicit ratings of the agent's atypicality^5^ and human similarity. *Pretesting only*: each trial was preceded by a 1 s fixation point and followed by a 2 s rest period.

To confirm that perception of the agents was as expected (e.g., atypical agents rated as high in atypicality, etc.), we first pretested these 120 photographs (20 agents per each of the six intended design conditions) with 30 participants (recruited from Tufts university and granted course credit in exchange for their participation). Participants were shown the 120 photographs sequentially and in an order randomized by participant. For each image, we measured the agent's “category ambiguity” (indexed by participants' accuracy in a categorization task and their latency to respond), atypicality, and human similarity (see Figure 1). Then, to concentrate atypicality within the set of atypical agents and category ambiguity within the set of ambiguous agents, we reduced the pretested set of 120 photographs down to 60 (with 10 instances per agent category) by selecting for category-ambiguous agents with lowest atypicality and atypical agents with lowest category ambiguity.

#### 2.2.3. Manipulation checks

To confirm that this final set of 60 images reflected our design assumptions (that agents labeled as atypical were perceived as most atypical, agents labeled as ambiguous were most ambiguous, etc.), analyses of variance (ANOVA) were conducted on the dependent variables indexing ambiguity (categorization error rate, response time) and atypicality with *typicality* as the independent variable. Each ANOVA revealing significant effects was followed by *t*-tests examining the planned, pairwise contrasts (*atypical, ambiguous* vs. *prototypic*)^6^.

ANOVAs on categorization error rate and response time confirmed a significant main effect of *typicality* on perceptions of agent ambiguity [*F*_*error*_ (1.15, 33.42) = 86.94, *p* < 0.01, $\eta_{p}^{2}=0.75$; *F*_*RT*_ (2, 58) = 10.89, *p* < 0.01, $\eta_{p}^{2}$ = 0.27], in which *ambiguous* agents elicited the greatest difficulty (*p* < 0.01) in categorization [*M*_*error*_ = 0.32, *SD* = 0.18; *M*_*RT*_ (2, 58) = 1.92s, *SD* = 1.08 s] relative to both agents with prototypic appearances (*M*_*error*_ = 0.01, *SD* = 0.03; *M*_*RT*_ = 1.11 s, *SD* = 0.40 s) and those categorized as atypical (*M*_*error*_ = 0.04, *SD* = 0.06; *M*_*RT*_ = 1.78 s, *SD* = 1.23 s). Similarly, an ANOVA on atypicality ratings confirmed a main effect of *typicality* [*F*_(2, 58)_ = 276.46, *p* < 0.01, $\eta_{p}^{2}=0.91$], in which the set of *atypical* agents received significantly higher ratings of atypicality (*M* = 4.59, *SD* = 1.00) relative to prototypic agents (*M* = 1.78, *SD* = 0.60; *p* < 0.01).

In addition, an ANOVA on ratings of human likeness confirmed a main effect of *human similarity* [*F*_(1.58, 45.80)_ = 512.97, *p* < 0.01, $\eta_{p}^{2}\;=\;0.95$], in which the highly humanlike (but not prototypically human) agents received significantly higher ratings (*M* = 7.02, *SD* = 0.84) than prototypic robots (*M* = 2.66, *SD* = 1.26; *p* < 0.01) and significantly lower ratings than prototypic persons (*M* = 8.91, *SD* = 0.22; *p* < 0.01).

### 2.3. Experiment

#### 2.3.1. Participants

Seventy-five new participants (participants who took part in pretesting were excluded from participating here) were recruited from Tufts University and the surrounding community (the Greater Boston Area), and received either course credit (*n* = 45) or monetary compensation (*n* = 30) at a rate of *$*10/h for their participation. Data were unavailable for three participants due to software crashes (*n* = 2) and termination of a session due to failure to follow instructions (*n* = 1). Thus, a total of 72 participants (26 male) with ages ranging from 18 to 49 years (*M* = 19.73, *SD* = 4.00) were included in our final sample.

#### 2.3.2. Procedure

**Figure 2:**
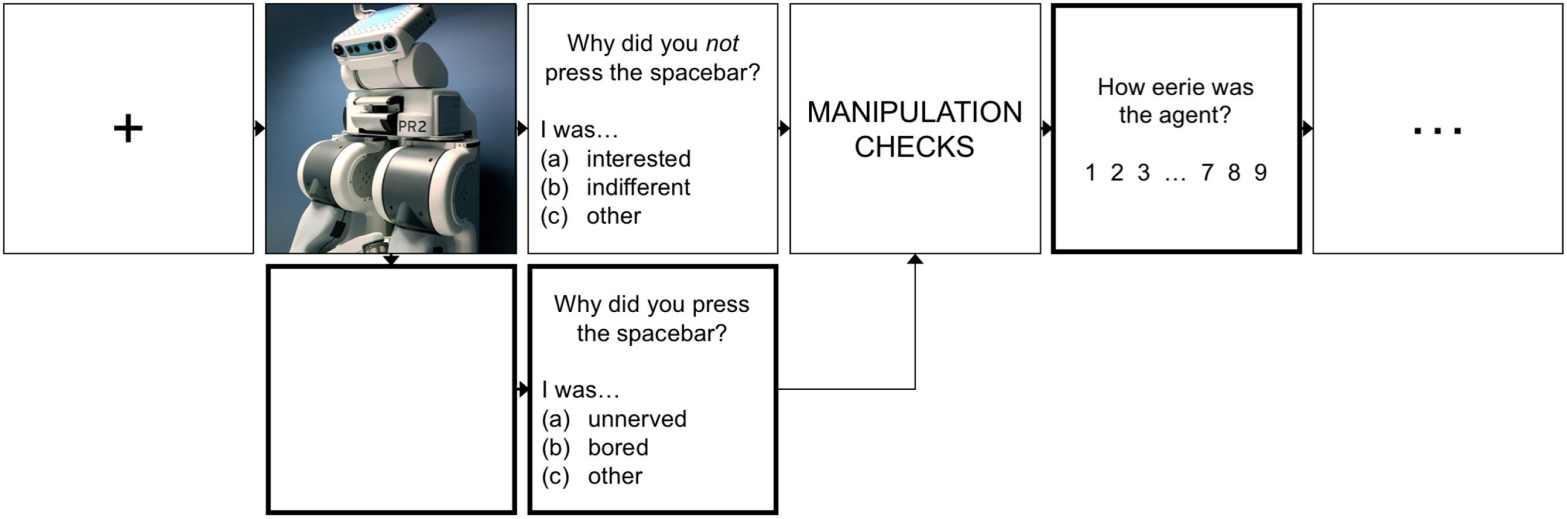
Trial Structure. Each trial began with a 1 s fixation point, followed by a picture viewing of up to 12 s. During the viewing, participants had the option to terminate the encounter early by pressing the spacebar. In doing so, the image was removed from display, leaving a blank screen for the remainder of the 12 s period. After the viewing, participants (1) were prompted for their reasons as to why they did or did not press the spacebar, (2) completed a series of checks to confirm whether the manipulations of agent appearance had the intended effects, and (3) provided an explicit rating of the agent's eeriness.

The final set of 60 photographs were shown using Processing 3.2.1 (©The Processing Foundation) in random order. Each trial began with a 1 s fixation point followed by the image presentation, and ended with a 2 s rest period (see Figure 2). During the viewing period, an image was presented for up to 12 s during which time participants had the option to press a button (the spacebar) to remove the image from the screen. If the participant did not press the spacebar, the image was shown for the full viewing duration (12 s). Otherwise, the image was removed as soon as participants pressed the spacebar, leaving a blank screen for the remainder of the viewing period^7^. After the viewing period, participants were prompted for their rationale as to why they terminated or did not terminate the encounter, followed by several manipulation checks (see Figure 1) and prompt for participants' explicit perceptions of the agent's eeriness. At the end of the picture-viewing protocol, participants were given a brief questionnaire to assess their attitudes toward robots.

#### 2.3.3. Measures

To index participants' aversion, we employed three primary measures derived from those developed in Strait et al. (2015):

- **Eeriness**: participants' subjective ratings of the agents' appearances. As we used a fully within-subjects design, ratings were averaged (by participant) across trials within each of the six agent categories.
- **Termination frequency**: the frequency at which participants elected to end their encounters with the various agents (computed within each of the six agent conditions as the proportion of trials in which participants pressed the spacebar to terminate the trial).
- **Terminations due to discomfort**: the proportion of terminated trials in which participants reported terminating due to being unnerved by the shown agent.

Finally, to index participants' attitudes toward robots, we used the Negative Attitudes Toward Robots Scale (NARS; Nomura et al., 2006). The scale is comprised of 14 questionnaire items and produces an overall NARS score (Cronbach's α = 0.87), as well as three subscores: negative attitude toward situations concerning interaction with robots (6 items; α = 0.78), negative attitude toward the social influence of robots (5 items; α = 0.70), and negative attitude toward emotions in interacting with robots (3 items; α = 0.77).

## 3. Results

### 3.1. Valley hypothesis (H1)

Based on Mori's uncanny valley theory, we hypothesized that—relative to robots of low human similarity and persons of prototypically human appearances—highly humanlike (but not prototypic) agents can be so discomforting that people would be averse to interacting with them. To test our hypotheses, a repeated-measures ANOVA was conducted on each of the three dependent variables and relevant manipulation check.^6^^,^ ^8^ All statistics (descriptive and inferential) are reported in Table 2, with effect sizes^9^ for significant contrasts reported in the discussion below.

**Table 2:** Main effects of the *human similarity* manipulation (within-subjects; three levels: low, high^10^, and prototypic) and corresponding descriptive statistics (means and standard deviation for each of the three levels).

	***n***	***DF*_*n*_**	***DF*_*d*_**	***F***	***p***	**$\eta_{p}^{2}$**	**Low**	**High**	**Prototypic**
**MANIPULATION CHECKS**									
Human Similarity Rating	72	1.55	110.26	1188.44	< 0.01	0.94	2.99 (1.16)	7.06 (0.81)	8.83 (0.33)
**HYPOTHESIS TESTING**									
Eeriness Rating	72	1.73	122.68	250.92	< 0.01	0.78	2.50 (1.37)	5.05 (1.13)	1.26 (0.69)
Termination Frequency	72	2	142	250.92	< 0.01	0.09	0.30 (0.37)	0.38 (0.35)	0.37 (0.38)
**Rationale for Terminating:**									
Unnerved	39	2	76	39.84	< 0.01	0.51	0.11 (0.29)	0.48 (0.33)	0.03 (0.11)
Bored	39	2	76	33.44	< 0.01	0.47	0.74 (0.39)	0.36 (0.33)	0.83 (0.32)
Other	39	2	76	0.18	0.83	0.00	0.14 (0.30)	0.16 (0.27)	0.14 (0.28)
**Rationale for Viewing:**									
Interested	58	2	114	25.38	< 0.01	0.31	0.52 (0.33)	0.62 (0.29)	0.32 (0.36)
Indifferent	58	1.79	102.12	30.01	< 0.01	0.34	0.45 (0.32)	0.32 (0.29)	0.64 (0.36)
Other	58	−	−	−	−	−	0.00 (0.00)	0.00 (0.00)	0.00 (0.00)

*Note that inferential statistics are unavailable for the “other” response rationale (for viewing), as the variance of the data was zero*.

#### 3.1.1. Manipulation check

We assumed that the three similarity designations—robots of low human similarity, highly humanlike agents, and people of prototypically human appearances—would be perceived as having monotonically increasing human similarity (from *low* to *prototypic*). To first confirm this assumption, we conducted an ANOVA on participants' ratings of the agents' human similarity with *human similarity* (*low, high, prototypic*) as the independent variable. As expected, the results showed a main effect of similarity ($\eta_{p}^{2}=0.94$). All pairwise contrasts were significant, with ratings increasing from robots designated as low in human similarity to highly humanlike agents (Cohen's *d*_*z*_ = 3.50) to people of prototypic similarity (*d*_*z*_ = 2.50).

#### 3.1.2. Hypothesis testing

**Figure 3:**
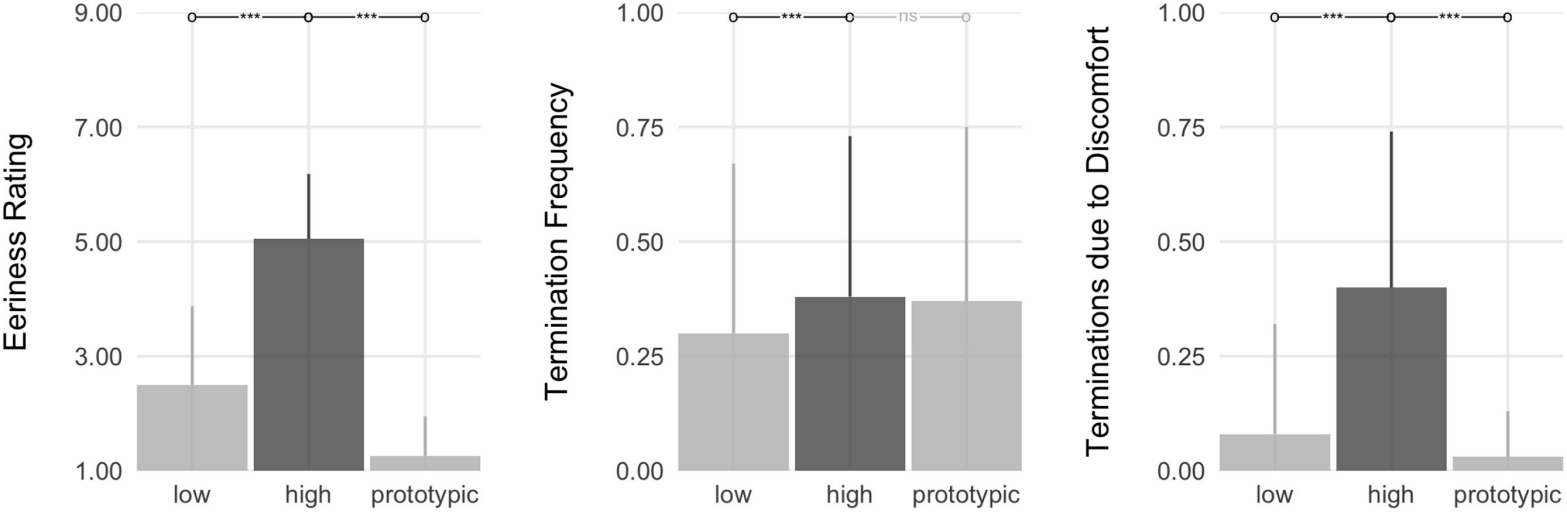
Test of the valley hypothesis (H1). Shown are the three indices of aversion by *human similarity* level: *low, high* (inclusive of both atypical and ambiguous robots and people), and *prototypic*. The bars show the planned contrasts, with asterisks denoting the significance level (^*^p < 0.05, ^**^p < 0.01, ^***^p < 0.001; or *ns*: nonsignificant).

We expected that, relative to both robots of low human similarity and persons of prototypically human appearances, participants would be averse to highly humanlike agents, evidenced by higher ratings of eeriness (**H1a**), more frequent termination of their encounters (**H1b**), and a greater proportion of terminated encounters terminated due to being unnerved (**H1c**). As expected, there was a main effect of *human similarity* on all three indices of aversion—eeriness ratings ($\eta_{p}^{2}=0.78$), termination frequency ($\eta_{p}^{2}=0.09$), and the frequency of terminations due to being unnerved ($\eta_{p}^{2}=0.51$) (see Figure 3).

Consistent with the valley theory, participants rated highly humanlike agents as eerier than both robots of low human similarity (*d*_*z*_ = 1.47) and prototypic persons (*d*_*z*_ = 2.93). In addition, they terminated encounters with highly humanlike agents more frequently than those with robots of low human similarity (*d*_*z*_ = 0.45). Lastly, although there was no significant difference in participants' termination frequency between encounters with highly humanlike agents vs. prototypic persons, significant differences did manifest in their rationale for terminating. Specifically, participants reported terminating encounters due to being unnerved more frequently in response to highly humanlike agents than they did in response to robots of less human similarity (*d*_*z*_ = 1.03) and prototypic persons (*d*_*z*_ = 1.29). For comparison, when participants terminated encounters with prototypic persons or with robots of low human similarity, their rationale for doing so stemmed largely from boredom (see Table 2).

Taken together, the results show strong support of Mori's valley hypothesis. Specifically, relative to robots of low human similarity and persons of prototypically human appearances, participants exhibited greater aversion (as evidenced by their eeriness ratings and avoidance rationale) toward highly humanlike—but not prototypically human—agents.

### 3.2. Mechanisms underlying uncanniness (M1–M2)

In identifying an uncanny valley in the current design space of humanoid robots and range of human appearances, we moved to testing the mechanisms underlying uncanniness. Here, we had hypothesized that both atypicality (M1: Feature Atypicality) and ambiguity (M2: Category Ambiguity) drive people's aversion toward highly humanlike (but not prototypically human) agents. Specifically, to understand *when/why/how* certain agents fall into the uncanny valley, we investigated two visual variables (atypicality, ambiguity) for their impact on people's perceptions of highly humanlike agents relative to agents of prototypic appearances.

In testing these hypotheses and corresponding assumptions, we ran 2 × 3 within-subjects ANOVAs with the IVs—*category* (two levels: *robot* and *person*) and *typicality* (three levels: *prototypic, atypical*, and *ambiguous*)—on each of the three indices of aversion.^6^^,^ ^8^ Note that, while we included *category* as an IV (due to its inclusion in the experimental design), both of the two mechanisms require that the valley effect is evident regardless of the agent's category membership. Thus, the testing of the two mechanisms relies on the main effect of *typicality*, not the *category* × *typicality* (which we explore later). To test the two mechanisms, we examined two *a priori* contrasts of interest as follows: *prototypic* vs. *atypical* (M1) and *prototypic* vs. *ambiguous* (M2). All statistics (descriptive and inferential) are reported in Table 3, with effect sizes^9^ for significant contrasts reported in the discussion below.

**Table 3:** Main effects of the *typicality* manipulation (within-subjects; three levels: prototypic^12^, atypical, ambiguous) and corresponding descriptive statistics (means and standard deviation for each of the three levels).

	***n***	***DF*_*n*_**	***DF*_*d*_**	***F***	***p***	**$\eta_{p}^{2}$**	**Prototypic**	**Atypical**	**Ambiguous**
**MANIPULATION CHECKS**									
Atypicality Rating	72	1.56	110.70	419.60	< 0.01	0.86	1.80 (.68)	4.25 (1.13)	5.75 (1.52)
Categorization Error (%)	72	1.09	77.64	111.68	< 0.01	0.61	0.00 (.01)	0.02 (0.04)	0.20 (0.15)
RT (*s*)	72	1.23	87.60	39.74	< 0.01	0.36	0.76 (0.51)	0.93 (0.65)	1.29 (0.98)
**HYPOTHESIS TESTING**									
Eeriness Rating	72	2	142	216.52	< 0.01	0.75	1.88 (0.85)	5.30 (1.33)	4.81 (1.34)
Termination Frequency	72	1.84	130.89	4.57	0.01	0.06	0.33 (0.36)	0.36 (0.35)	0.40 (0.36)
**Rationale for Terminating:**									
Unnerved	23	1.51	33.25	21.76	< 0.01	0.50	0.04 (0.12)	0.37 (0.34)	0.44 (0.37)
Bored	23	1.02	22.37	21.85	< 0.01	0.50	0.78 (0.33)	0.45 (0.37)	0.38 (0.38)
Other	23	2	44	1.52	0.22	0.06	0.17 (0.29)	0.21 (0.31)	0.17 (0.28)
**Rationale for Viewing:**									
Interested	52	1.52	77.30	29.61	< 0.01	0.37	0.42 (0.28)	0.67 (0.28)	0.55 (0.32)
Indifferent	52	1.68	85.45	38.37	< 0.01	0.43	0.55 (0.29)	0.28 (0.29)	0.39 (0.32)
Other	52	−	−	−	−	−	0.00 (.00)	0.00 (0.00)	0.00 (0.00)

*Inferential statistics are unavailable for the “other” response rationale (for viewing), as the variance of the data was again zero*.

#### 3.2.1. Manipulation checks

Here we made two additional assumptions in our experimental design. First, we expected that the agents categorized as *atypical* would be perceived as more atypical than the other typicality conditions (prototypic, ambiguous). As expected, an ANOVA on atypicality ratings showed a main effect of *typicality* condition ($\eta_{p}^{2}=0.86$). Contrary to our expectations, however, the post hoc contrasts showed *ambiguous* agents to prompt the highest ratings of atypicality, followed by atypical agents (*d*_*z*_ = −1.23), and lastly, prototypic agents (*d*_*z*_ = 2.79). A significant interaction with *ontological category* ($\eta_{p}^{2}\;=\;0.79$) confirmed our assumption with respect to the robotic agents. Specifically, atypical robots elicited the highest ratings of atypicality relative to both prototypic (*d*_*z*_ = 2.77) and ambiguous robots (*d*_*z*_ = 0.91). Whereas, amongst human agents, persons of ambiguous category membership elicited higher ratings than persons with atypical features (*d*_*z*_ = −2.25)^11^. Nevertheless, participants rated persons with atypical features as more atypical than prototypic persons (*d*_*z*_ = 0.97).

Second, we assumed the *ambiguous* agents (agents proximate to a nonhuman–human category boundary) would elicit difficulty in deciding their category membership (robot or person) on a categorization task. Furthermore, we assumed categorization difficulty would be reflected by participants' error in categorizing and latency to respond (RT). As expected, there was a main effect of *typicality* on both categorization error ($\eta_{p}^{2}=0.61$) and RT ($\eta_{p}^{2}=0.36$). Specifically, ambiguous agents elicited greater categorization error and longer response times in categorizing relative to both prototypic (*d*_*error*_ = 1.29; *d*_*RT*_ = 0.81) and atypical agents (*d*_*error*_ = 1.22; *d*_*RT*_ = 0.65).

#### 3.2.2. Hypothesis testing

**Figure 4:**
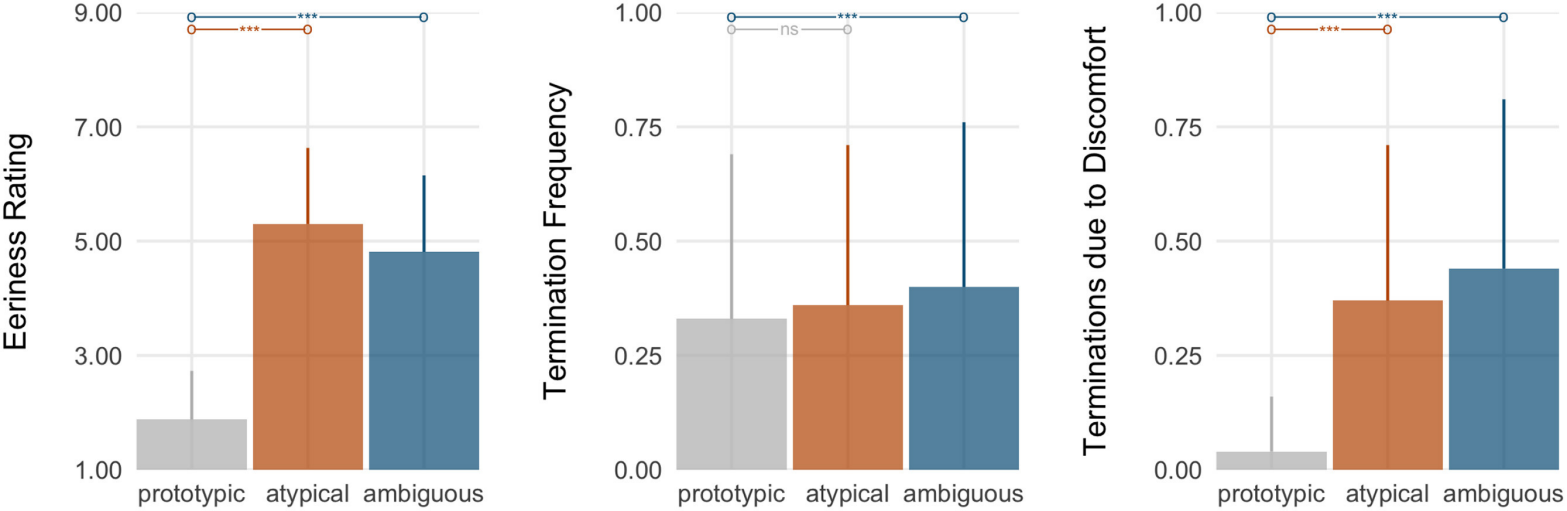
Test of the underlying mechanisms (M1–M2). Shown are the main effects of *typicality* (prototypic, atypical, and ambiguous) on the three indices of aversion. Bars show the planned contrasts, with asterisks denoting significance.

We had hypothesized that, relative to agents of prototypic appearances, agents with *feature atypicality* (**M1**) and *category ambiguity* (**M2**) would elicit aversion in participants. Consistent with our predictions, the results show a main effect of *typicality* on the three indices of aversion: eeriness ratings ($\eta_{p}^{2}=0.75$), termination frequency ($\eta_{p}^{2}=0.06$), and the frequency of terminations due to being unnerved ($\eta_{p}^{2}=0.50$) (see Figure 4).

Specifically, the planned contrasts show that participants rated both atypical and ambiguous agents as significantly eerier than prototypic agents (*d*_*atypical*_ = 2.07; *d*_*ambiguous*_ = 2.02). In addition, when participants terminated encounters with atypical and ambiguous agents, they did so more frequently due to being unnerved (*d*_*atypical*_ = 1.01; *d*_*ambiguous*_ = 1.13) than they did in response to prototypic agents. For comparison, when participants terminated encounters with prototypic agents, their rationale for doing so stemmed largely from boredom (see Table 3).

However, only agents with ambiguous appearances prompted more frequent avoidance. Specifically, participants terminated encounters with ambiguous agents more frequently than they did with prototypic agents (*d*_*z*_ = 0.31).

In sum, the results here show support for both theoretical accounts (feature atypicality and category ambiguity). Specifically, consistent with **M1** (*feature atypicality*), participants rated atypical agents as eerier than prototypic agents and avoided them more frequently due to being unnerved. Similarly, consistent with **M2** (*category ambiguity*), participants rated ambiguous agents as eerier than prototypic agents, avoided them more frequently, specifically due to being unnerved.

#### 3.2.3. Secondary analyses

**Figure 5:**
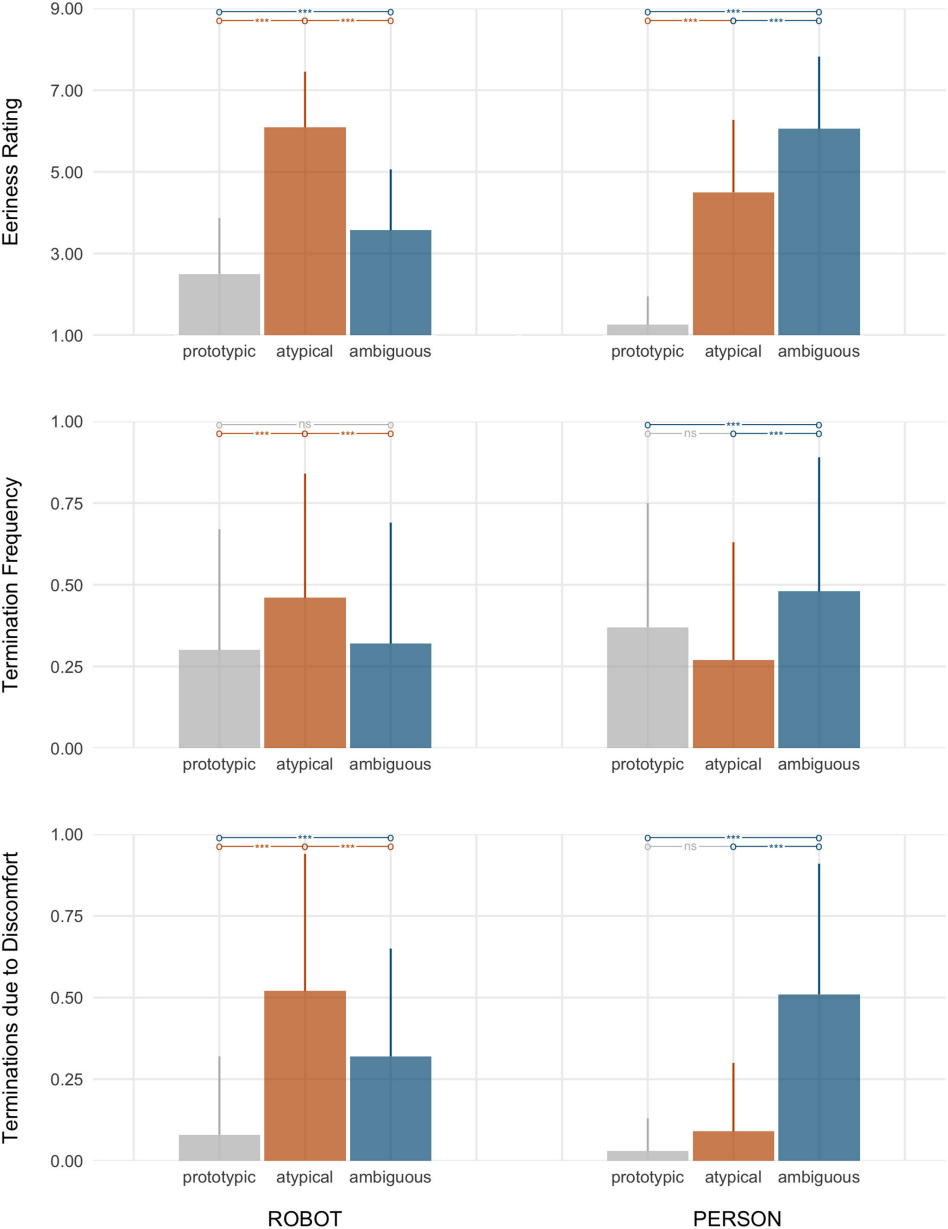
Participants' aversive responding (top: eeriness; middle: termination frequency; bottom: proportion of terminations due to being unnerved) by *typicality* (prototypic, atypical, and ambiguous) and within agent *category* (left grouping: robot; right grouping: person).

While we found support for both theoretical mechanisms, we also observed a significant interaction between the agents' ontological *category* and *typicality* on eeriness ratings ($\eta_{p}^{2}\;=\;0.71$), termination frequency ($\eta_{p}^{2}=0.34$), and the frequency of termi-nations due to being unnerved ($\eta_{p}^{2}=0.59$), thus indicating that the effect of typicality manifests differently depending on whether the agent in question is a robot or a human (see Figure 5). Hence, we proceeded to explore the pairwise contrasts between typicality levels and agent category.

Note that the exploration, however, was limited to *within* the respective agent category. This follows from the theoretical motivations for investigating feature atypicality (M1) and category ambiguity (M2) for their role in the valley effect, which rely on contrasts between agents of prototypic appearances relative to those of atypic and ambiguous appearances. Here, we also explored the contrast between agents of atypic vs. ambiguous appearances toward understanding whether one vs. the other more strongly provokes discomfort.

##### 3.2.3.1. Responding toward robots

Across all three measures of interest (eeriness ratings, termination frequency, and terminations due to being unnerved), the within-category pairwise contrasts suggest that *atypicality* drove participants' aversion toward robots (see Figure 5, top).

Prototypic robots, as expected, were rated as the least eerie of all robot stimuli. In addition, participants terminated their encounters less frequently, and when they did so, it was rarely due to being unnerved (see Table 4). On the other end, atypical robots—relative to *both* prototypic and ambiguous robots—were rated as most eerie (*d*_*z*_ = 1.94; *d*_*z*_ = 1.48). Participants also terminated encounters with atypical robots at the highest frequencies (*d*_*z*_ = 0.59; *d*_*z*_ = 0.63), and did so most frequently due to being unnerved (*d*_*z*_ = 1.07; *d*_*z*_ = 0.90). Participants did also exhibit aversion to interacting with ambiguous robots (though less so than their aversive responding toward the set of atypical robots). Specifically, participants rated ambiguous robots as more eerie (*d*_*z*_ = 0.63) and terminated their encounters more frequently due to being unnerved (*d*_*z*_ = 0.73) relative to prototypic robots. Surprisingly, however, participants were not any more avoidant (evidenced by the frequency at which participants' terminated their encounters) of ambiguous robots than they were of prototypic robots.

**Table 4:** Interaction between the *typicality* × *ontology* manipulations, as well as the corresponding descriptive statistics (means and standard deviation) for each of the three typicality conditions (prototypic, atypical, and ambiguous) by category membership (top: robot; bottom: person).

	***n***	***DF*_*n*_**	***DF*_*d*_**	***F***	***p***	**$\eta_{p}^{2}$**	**Prototypic**	**Atypical**	**Ambiguous**
**MANIPULATION CHECKS**									
Atypicality Rating	72	1.80	127.53	260.84	< 0.01	0.79	2.37 (1.22)	6.30 (1.66)	5.01 (1.72)
							1.22 (0.34)	2.20 (1.05)	6.49 (1.80)
Categorization Error (%)	72	1.04	74.11	4.71	0.03	0.06	0.00 (0.01)	0.03 (0.06)	0.26 (0.27)
							0.00 (0.02)	0.01 (0.04)	0.14 (0.27)
RT (*s*)	72	1.35	95.63	1.42	0.24	0.02	0.76 (0.53)	0.90 (0.63)	1.35 (1.07)
							0.77 (0.52)	0.95 (0.72)	1.24 (1.09)
**HYPOTHESIS TESTING**									
Eeriness Rating	72	2	142	177.61	< 0.01	0.71	2.50 (1.37)	6.09 (1.36)	3.57 (1.49)
							1.26 (.69)	4.50 (1.77)	6.05 (1.77)
Termination Frequency	72	1.68	119.18	37.06	< 0.01	0.34	0.30 (0.37)	0.46 (0.38)	0.32 (0.37)
							0.37 (0.38)	0.27 (0.36)	0.48 (0.41)
**Rationale for Terminating:**									
Unnerved	23	2	44	32.04	< 0.01	0.59	0.08 (0.24)	0.52 (0.42)	0.32 (0.33)
							0.03 (0.10)	0.09 (0.21)	0.51 (0.40)
Bored	23	2	44	14.39	< 0.01	0.40	0.75 (0.38)	0.31 (0.40)	0.44 (0.38)
							0.81 (0.32)	0.66 (0.39)	0.37 (0.40)
Other	23	2	44	5.41	< 0.01	0.20	0.17 (0.31)	0.18 (0.31)	0.24 (0.31)
							0.16 (0.28)	0.25 (0.36)	0.12 (0.28)
**Rationale for Viewing:**									
Interested	52	2	102	12.05	< 0.01	0.19	0.50 (0.33)	0.61 (0.35)	0.65 (0.32)
							0.31 (0.35)	0.69 (0.29)	0.42 (0.41)
Indifferent	52	2	102	7.83	< 0.01	0.13	0.47 (0.32)	0.30 (0.35)	0.29 (0.31)
							0.64 (0.35)	0.28 (0.29)	0.49 (0.40)
Other	52	−	−	−	−	−	0.00 (0.00)	0.00 (0.00)	0.00 (0.00)
							0.00 (0.00)	0.00 (0.00)	0.00 (0.00)

##### 3.2.3.2. Responding toward people

Similar to prototypic robots, persons of prototypic appearances were rated as the least eerie of all persons depicted. Furthermore, though participants terminated approximately a third of their encounters with prototypic persons, when they did so, it was again rarely due to being unnerved (see Table 4). In contrast, however, to participant responding toward non-prototypic robots (in which atypicality provoked the greatest aversion), category ambiguity appeared to drive participants' aversion toward the human stimuli (see Figure 5, bottom). Specifically, participants rated persons of ambiguous category membership as most eerie, relative to both prototypic persons (*d*_*z*_ = 2.54) and persons with atypical features (*d*_*z*_ = −0.84). They also terminated their encounters with ambiguous persons at the highest frequencies (*d*_*z*_ = 0.38; *d*_*z*_ = −0.70), and did so most frequently due to being unnerved (*d*_*z*_ = 1.21; *d*_*z*_ = −1.22). In fact, participants terminated their encounters with persons with atypical features significantly *less* frequently than their encounters with prototypic persons (*d*_*z*_ = −0.43) and there was no significant difference between atypical and prototypic persons in the proportion of encounters that they terminated due to being unnerved.

Overall, the secondary analyses reveal that the data reflect greater support for the feature atypicality hypothesis with respect to robotic agents. With respect to human agents, the results are suggestive of greater support for the category ambiguity hypothesis, but uncertainty arising from the study's manipulation checks warrants further investigation of this finding^13^.

### 3.3. Negative attitudes toward robots

Lastly, we explored whether participants' aversive responding toward our stimuli could be explained by pre-existing negative attitudes about robots. Using the Negative Attitudes toward Robots Scale, we tested participants' overall NARS score and scores on the three NARS subscales – negative attitude toward situations concerning interactions with robots (S1), negative attitude toward the social influence of robots (S2), and negative attitude toward emotions in interacting with robots (S3) – for any relationship to their subjective and behavioral responding on the three indices of aversion (eeriness ratings, termination frequency, terminations due to being unnerved). For each aversion index, we computed participants' average response toward all robotic stimuli and by category (prototypic, atypical, ambiguous). In total, we computed 48 correlations (three NARS subscales, plus an overall NARS score; three agent categories, plus an overall response; three aversion indices) using Pearson's *r* test (see Table 5). However, no significant relationships were found.

**Table 5:** Correlation matrix between the NARS scales (overall score and by subscales: negative attitude toward situations concerning *interactions with robots*; negative attitude toward the *social influence of robots*; and negative attitude toward *emotions* in interacting with robots) and participants' responding toward robots (overall and by category – prototypic, atypical, and ambiguous) on the three indices of aversion (eeriness rating, termination frequency, and proportion of terminations terminated due to being unnerved).

	**NARS**	**Interactions with robots**	**Social influence of Robots**	**Emotions in interactions**
Eeriness Rating	0.14	0.13	0.17	0.04
Prototypic	0.17	0.13	0.16	0.15
Atypical	0.01	−0.05	0.02	0.09
Ambiguous	0.10	0.18	0.15	−0.14
Termination Frequency	0.13	0.12	0.11	0.08
Prototypic	0.16	0.12	0.13	0.16
Atypical	0.08	0.14	0.05	−0.02
Ambiguous	0.11	0.07	0.13	0.09
Unnerved Rationale	0.09	0.20	0.00	−0.06
Prototypic	0.09	0.21	−0.04	−0.01
Atypical	0.04	0.12	−0.04	−0.03
Ambiguous	0.10	0.25	−0.02	−0.07

*No significant correlations exist*.

## 4. Discussion

In the nearly 50 years since Mori's formalization of the uncanny valley (Mori et al., 2012), substantial empirical support has been found for the hypothesis that agents with highly humanlike (but not prototypically human) appearances provoke aversive responding in observers (Kätsyri et al., 2015; Rosenthal-von der Pütten and Krämer, 2015; MacDorman and Chattopadhyay, 2016). Yet, the mechanisms that lead to such feelings of discomfort are largely unknown. Moreover, many still question whether a valley even exists (e.g., Brenton et al., 2005; Hanson et al., 2005; Bartneck et al., 2009; Burleigh et al., 2013; Zlotowski et al., 2013; Złotowski et al., 2015).

Those questioning uncanny valley theory are not wrong: evidence of the valley effect is not in overabundance and the evidence which does exist varies widely in methodologies used (Kätsyri et al., 2015), leaving numerous gaps in the literature. In particular, much of the valley literature is based on (1) stimuli that represent a small subset of a large design space (humanoid robots) and (2) measures that do not capture behavioral implications (relying instead on explicit perception). Thus, the questions of whether the valley effect is robust (i.e., does it generalize to the broader design space) and relevant to human-robot interaction remain.

Two recent studies, using the largest stimulus sets to date (45-80 robots), suggest that the valley effect is both robust and profoundly impactful (Strait et al., 2015; Mathur and Reichling, 2016). Specifically, using picture-based methodologies and behavioral measures to supplement the traditional metrics, the two studies evaluated the impact of a robot's appearance on people's behavior toward a broad range of humanoid robots. In particular, Mathur and Reichling (2016) found that the valley reduces people's trust in highly humanlike robots and we (Strait et al., 2015) found that, not only do people dislike highly humanlike robots, but people actively avoid interacting with them.

As a test of its replicability and extension to this recent work, we adapted the methodologies of Mathur and Reichling (2016) and Strait et al. (2015) for another experimental investigation of the valley's existence and the design factors that underlie uncanniness. In particular, we tested two theoretically-motivated factors—atypicality and category ambiguity—for their effects on perceptions of uncanniness and resulting avoidant behaviors. Furthermore, we tested an outstanding and common critique of the valley—namely, whether people's aversive responding can be alternatively explained by pre-existing negative attitudes toward robots.

### 4.1. Summary of findings

#### 4.1.1. Replication of the valley effect (H1)

Consistent with our expectations and previous literature (e.g., MacDorman, 2006; Kätsyri et al., 2015; Strait et al., 2015; Mathur and Reichling, 2016), participants exhibited clear aversion toward agents of high human similarity (highly humanlike robots and humans with non-prototypic appearances), as evidenced by higher ratings of eeriness, more frequent avoidance (early termination of their encounters^14^), and more frequent termination due to being *unnerved*.

While there was not a significant difference in termination frequencies between agents of high similarity and (prototypic) humans, participants' endorsed different rationales for terminating these encounters. Specifically, participants terminated encounters with human stimuli largely due to boredom. By contrast, participants terminated over a third of their encounters with highly humanlike agents due to being unnerved. In particular, it is worth noting that, while the stimuli used in the present study were both innocuous and fleeting, participants nevertheless exhibited significant aversion in their encounters with the highly humanlike agents. That is, the appearances of the highly humanlike agents was discomforting enough that participants often preferred to look at a blank screen, rather than the agents themselves.

Beyond the confirmation of our first hypothesis, the data here fully replicate and thus validate the findings of Strait et al. (2015), demonstrating empirically that the uncanny valley—as a function of human similarity—provokes robust, emotionally-motivated responses to humanlike robots. Our results also lend further support to the findings by Mathur and Reichling (2016) that robots with highly humanlike appearances profoundly (and negatively) impact people's behavioral responding.

#### 4.1.2. Understanding the uncanny (M1, M2)

As hypothesized and consistent with prior indications (Mitchell et al., 2011; Chattopadhyay and MacDorman, 2016; MacDorman and Chattopadhyay, 2016), *atypicality* provoked aversive responding relative to agents with more typical appearances as evidenced by participants' ratings of the agents' eeriness and the proportion of encounters terminated early due to being unnerved (M1). Support was also found for the hypothesized effect of category *ambiguity* (M2). Specifically, similar to participants' responding toward atypical agents, participants exhibited significant aversion toward agents of ambiguous category membership relative to prototypic agents as evidenced by all three indices of aversion (respective eeriness ratings, termination frequency, and proportion of encounters terminated due to being unnerved).

Exploration of the *typicality* × *category* interaction, however, suggests that the mechanisms have differential impact on responding depending on whether the agent in question is robot or human. Specifically, within the set of robotic stimuli, atypicality provoked the greatest aversion (highest ratings of eeriness, more frequent termination of encounters, and greatest proportion of encounters terminated due to being unnerved). In fact, while the set of ambiguous robots – relative to prototypic robots – prompted higher eeriness ratings and more encounters to be terminated due to being unnerved, they did not elicit greater avoidance (there was no significant difference in the termination frequency from that in response to prototypic robots). Moreover, the ambiguous stimuli were neither the eeriest nor the most discomforting.

In contrast, within the set of human agents, ambiguity provoked the greatest aversion in participants (higher ratings of eeriness, more frequent termination of encounters, and greater proportion of encounters terminated due to being unnerved). Surprisingly, while participants rated atypical stimuli as eerier than persons of prototypically human similarity, participants terminated their encounters with atypical stimuli *less* frequently than with ambiguous and prototypic stimuli.

#### 4.1.3. Negative attitudes toward robots

Exploration of alternative explanations of the above findings did *not* yield support for the suggestion that people's behavior may be explained by pre-existing and negative attitudes toward robots (rather than as the result of an uncanny valley phenomenon). Specifically, no significant relationships were found in 48 correlational tests between participants' aversion and their attitudes toward robots, as indexed by the NARS scales. These findings suggest that positive exposure and/or additional experience with robots is unlikely to affect the occurrence of an uncanny valley effect in humanoid robotics.

### 4.2. Implications

The present research has three primary theoretical and practical implications.

#### 4.2.1. Methodological practices

We validated a simple – but effective – laboratory procedure for assessment of people's aversion to social robots. In particular, we adapted a standard procedure from psychology research for the measurement of social signals (particularly, the experience and regulation of negative emotion) in laboratory-based human-robot interactions. The protocol contributes both instrumentation (the measurement of emotion-related social signals in HRI contexts), as well as an effective work-around for a longstanding methodological limitation (accessibility of physical robotic platforms).

Consistency across the multiple measures (of participants' emotion experience and emotionally-motivated responding) and between studies (Strait et al., 2015 and here) demonstrates the reliability of this approach. Whether and how these results transfer to more ecologically valid contexts (e.g., actual human-robot interaction in the wild) remains to be investigated. However, at a minimum, the protocol provides a means of making systematic probes of the various visual variables present in an agent's appearance.

#### 4.2.2. Uncanny valley theory

In providing another experimental test of the uncanny valley hypothesis, our study reveals a robust uncanny valley in the design space of social robots in terms of people's attribution of eeriness to highly humanlike (but not prototypically human) agents. More importantly, it validates the previously suggested (cf. Strait et al., 2015) link between avoidant behavior (early termination of encounters due to being unnerved) and highly humanlike robots. Furthermore, this work extends Mori's initial postulations to consider specific visual aspects that lead to uncanniness. Specifically, the findings point to both atypicality and category ambiguity as driving forces in people's discomfort. The two visual variables (atypicality and category ambiguity) resulted in higher ratings of eeriness, more frequent terminations of encounters, and a greater proportion of terminations terminated due to being unnerved.

**Figure 6:**
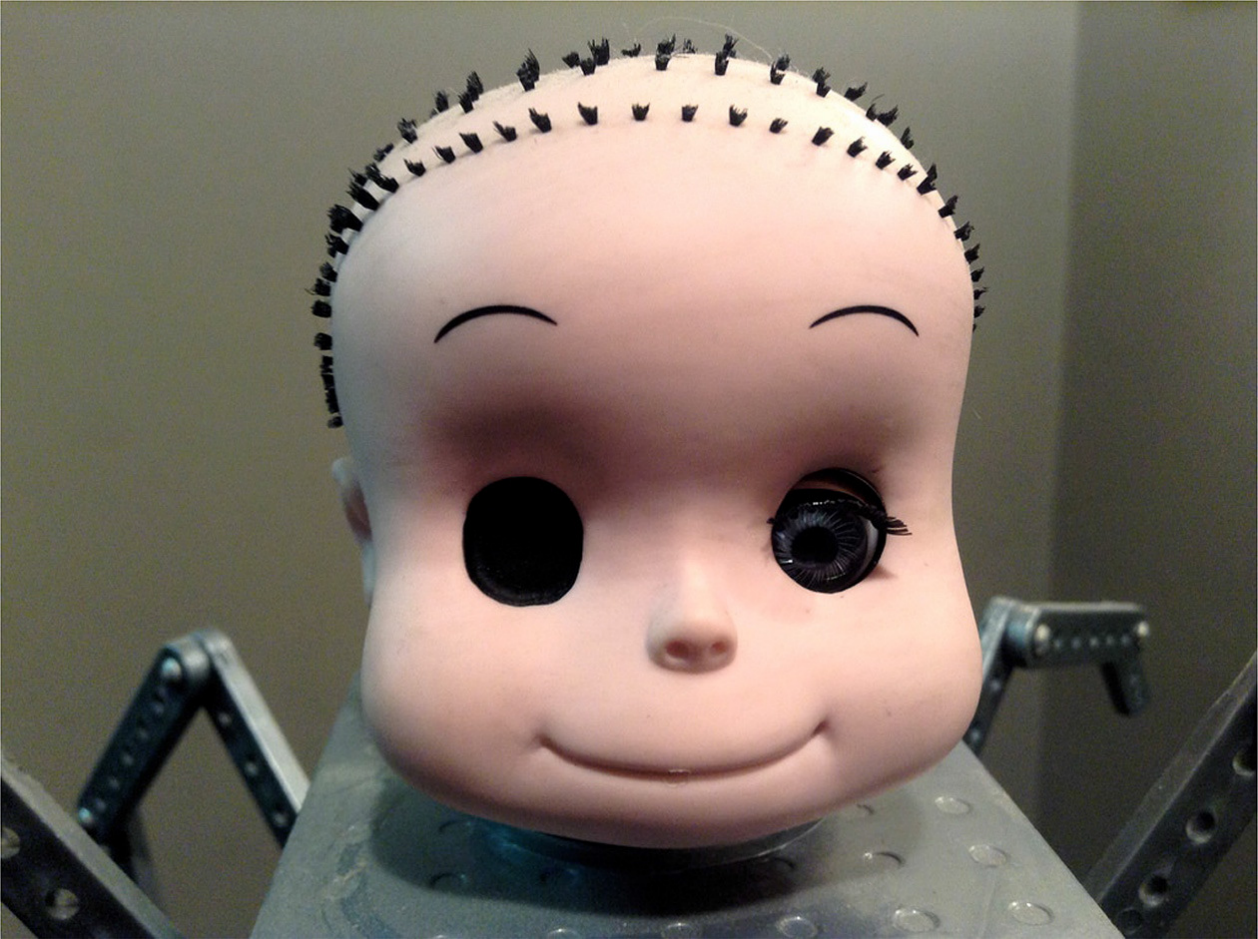
“Babyface” from Pixar's Toy Story (1995). Attribution: photograph (https://goo.gl/GkuBLQ) by Mike Mozart, available under a Creative Commons Attribution 2.0 Generic license1.

Of particular note, our exploratory analyses showed that the atypical robots (which were atypical in the combination of a highly humanlike head atop a mechanomorphic body) and ambiguous humans (which were dehumanized via the use of black, full-sclera contacts, thus occluding the iris) elicited the greatest aversion. These findings are consistent with prior literature evaluating *mind*-related (features related to the head, and in particular, the eyes) atypicalities (Gray and Wegner, 2012; Schein and Gray, 2015; Appel et al., 2016). In addition, the findings support the (relatively common) use of certain visual effects in media and film to instill a sense of unease in observers. Consider for example: Pixar's Babyface (see Figure 6) who was an unnerving (albeit eventually sympathetic) character in *Toy Story* (1995); Joshu Kasei, an ultimately terrifying character in *Psycho-Pass* (2012–); and the generally unsettling Ava in *Ex Machina* (2015), amongst others.

#### 4.2.3. Design considerations

Correspondingly, the findings here provide soft guidelines for the design of future humanoid systems. Participants' strong negative responding—particularly their frequent avoidance of encounters due to discomfort—establishes a shortcoming of the current design space. Moreover, the lack of any predictive relationship between participants' preexisting attitudes toward robots (as indexed by NARS) and their aversive responding suggests that the valley effect is not learned (e.g., via negative portrayals of robots in media) and furthermore, unlikely to dissipate with time/exposure. Thus, there is a clear need to consider alternatives to blanket anthropomorphization.

Broadly, participants' consistent aversion to highly humanlike robots demonstrates a significant cost to designing robots with high human similarity in their appearance. Our results do show evidence of increased interest in the robots corresponding to increased human similarity (consistent with the empirical motivations for increasingly anthropomorphized robot designs; e.g., Riek et al., 2009). However, the increase in interest we've observed pales in magnitude relative to the corresponding increase in avoidance due to discomfort. Moreover, despite significantly increased interest and stimuli that were both innocuous and fleeting, we have consistently observed participants' *avoidance* of encounters with (*photographs* of) highly humanlike robots. In considering that such aversion can be elicited in these settings and in spite of increased interest, we suggest that designing robots with *less* human similarity (at least in their appearance) is a practical and fast solution to the issues underscored by the present findings.

That being said, our results do not suggest that efforts to design humanlike robots are futile. Rather, they hint that attention to certain attributes when designing humanlike robots may mitigate aversive responding. Specifically, we note that the set of atypical robots provoked the greatest aversion in participants, more so than the set of “ambiguous” robots (androids). This finding is consistent with prior indications that androids do not necessarily elicit the most negative reactions (e.g., Rosenthal-von der Pütten and Krämer, 2014), and further, suggests that the valley effect can be attenuated, if not overcome. Thus, when designing humanlike robots, our data indicate that greater consistency amongst features may avoid the elicitation of aversion. For example, a prototypically mechanical body should be accompanied by a prototypically mechanical head, even if it means forgoing more humanlike features. Conversely, a highly humanlike head should be accompanied by a highly humanlike body.

### 4.3. Limitations and future directions

The present study contributes a replication and extension of prior research on the uncanny valley in the domain of social robotics and human-robot interaction. In particular, it demonstrates the use of a simple laboratory procedure to evaluate aversive responding with a large portion of the current design space of humanoid robots. While we are confident that the present study was well-suited to address our primary goals, the work also has its limitations that underscore important avenues for future research.

#### 4.3.1. Demographics

One potentially significant limitation in particular concerns the demographics of both our participants and of the humanlike stimuli employed in this study. Specifically, our sampling – despite attempts to recruit broader participation via public advertisement within the local metropolitan area – drew a largely homogenous (predominately white, well-educated, American, and young) participant population. While these demographics reflect those of the local university and to some extent, the geographical region in which the study was conducted, it nevertheless constrains the interpretation of our results. In particular, it remains unknown as to whether the observed valley effects extend to the general population as variations in participant demographics (e.g., age, culture, etc.) have been found to affect people's general perceptions of social robots (e.g., Bartneck et al., 2005; Kuo et al., 2009; Li et al., 2010; Lee and Sabanović, 2014; Stafford et al., 2014b; Sundar et al., 2016). Though these variations have not been studied directly in relation to the uncanny valley, still there may be a multitude of sociocultural factors relevant to understanding the valley phenomenon and its effects on the perception of and emotionally-motivated responding to robots.

In addition, it is important to note the simultaneous imbalance in the race/gender of our stimuli. Specifically, the set of highly humanlike robots is primarily composed of robots that are female-gendered and phenotypically Asian, while robots with lesser degrees of human similarity lack explicit race and gender cues. This imbalance stems from the “demographics” of the current design space of android robots, in which a majority of platforms have been modeled after women (who are predominately Asian) and white men. Though we balanced our set of human stimuli to reflect the demographics of the highly humanlike robots, the skewed demographics of both our stimuli and the participants evaluating it leave the potential for differential responding on the basis of the agents' gender/race (e.g., Fiske et al., 2007; Zebrowitz and Montepare, 2008). This thus poses a methodological consideration that warrants further investigation.

#### 4.3.2. Instrumentation

In addition to the above considerations, we also note a potential limitation with respect to the measurement of negative attitudes toward robots. Specifically, we employed the NARS scales (Nomura et al., 2006) for indexing participants' attitudes in order to address a longstanding critique of valley theory, namely whether people's aversion stems from pre-existing negative attitudes. Though no significant relationship was observed between the NARS and aversion indices, it is possible that the NARS scales do not capture negative attitudes that are relevant to the uncanny valley. Specifically, the content of the NARS questionnaire items range from context-related (e.g., “I would feel nervous just standing in front of a robot”) to highly philosophical in nature (e.g., “I would feel uneasy if robots really had emotions,” “I am concerned that robots would be a bad influence on children,” “I feel that in the future, society will be dominated by robots.”). Thus, the scale may align more with attitudes pertaining to human identity and replacement by robots (e.g., MacDorman, 2006; Rosenthal-von der Pütten and Krämer, 2015), which may not drive the behavioral valley effects observed here.

#### 4.3.3. Development

Finally, the majority of literature probing the valley and its effects is limited to young adults. Thus, it remains to be determined as to when/how the uncanny valley emerges over development. Specifically, are the indices of aversion that we observed here present in infants/children in a qualitatively similar way? Or is the valley limited to adults? While there is evidence of valley effects in infants (Lewkowicz and Ghazanfar, 2012; Matsuda et al., 2012), it is methodologically limited. In particular, the valley effects in infants are evidenced only by their gaze behavior and only in response to a very small set of agents. Additional studies evaluating valley effects in children would be useful both theoretically and practically. Theoretically, observation of a valley before young adulthood would lend support to the notion that the valley stems from more intrinsic perceptual mechanisms (e.g., the category uncertainty hypothesis and categorization theory). Practically, regardless of its innateness, understanding how younger populations perceive social robots would determine whether their design needs to be modified as a function of age of the population for which the robot is designed.

## 5. Conclusions

Our results both replicated and extended prior research, providing further empirical support for Mori's uncanny valley hypothesis and its relevance to human-robot interaction. Specifically, we demonstrated a robust valley effect within the current design space of humanoid robotics, wherein people showed significant behavioral aversion to highly humanlike robots. Moreover, we found no relationship between people's aversion and any pre-existing attitudes toward robots, suggesting that time and/or exposure to robots is unlikely to mitigate the valley effect. These findings underscore both a need for careful attention to the appearance of humanoid robots and the importance of measuring people's emotional responses to robots during the design phase.

At present, the findings serve to provide general guidance in the design of future social robots. In particular, our exploration points to two visual factors that should be considered, namely atypicality and category ambiguity. Our results suggest, for example, that it would be wise to design new robots with greater consistency between features and greater distance from the robot-human boundary (in either direction). Doing so may help to mitigate aversive reactions and, thus, maximize the utility of robots in contexts requiring interaction with humans.

## Ethics statement

All subjects gave written informed consent in accordance with the Declaration of Helsinki. The protocol was approved by the Tufts University Institutional Review Board.

## Author contributions

MS and HU conceived and designed the study, with significant input from VF, MJ, WJ, KM, and JR. MS conducted the data acquisition. MS analyzed the data, with significant input from HU, VF, MJ, WJ, KM, and JR. MS and HU wrote the manuscript, with significant input from VF, MJ, WJ, KM, and JR.

### Conflict of interest statement

The authors declare that the research was conducted in the absence of any commercial or financial relationships that could be construed as a potential conflict of interest.

## Footnotes

^1^In contrast to the aforementioned studies (which involved 1-3 robots), both studies referenced here involved 45–80 robots.

^2^Due to the current design space of humanoid robotics and range of human appearances (e.g., there do no exist stimuli depicting people of “low human similarity”), the study did not involve a factorial design.

^3^https://goo.gl/eTRg2B

^4^The current design space of highly humanlike robots is largely comprised of robots that are gendered/racialized (designed with physical features that convey gender/race) and skewed toward appearances that are female and Asian

^5^The atypicality prompt in Figure 1 is shortened from: “How *mismatched* are this agent's features relative to its overall appearance?” due to space constraints.

^6^All analyses were run in R (Version 3.3.1), with statistical significance defined as α = 0.05. For each ANOVA, the assumption of equal variance was confirmed using Mauchly's test of sphericity. In cases of violation, the reported degrees of freedom and corresponding *p*-value reflect a Greenhouse-Geisser adjustment as per Girden (1992). For the pairwise contrasts, two-tailed (rather than one-tailed) t-tests were used to reveal if/when a contrast went in the direction opposite to that which was predicted and reduce the overall rate of false positive results. Additionally, all pairwise contrasts reflect a Bonferroni correction for multiple comparisons. Lastly, note that while we defined statistical significance at α = 0.05, all significant results (including both tests of the hypotheses and Bonferroni-corrected contrasts) have a *p*-value of ≤.01 except where explicitly stated otherwise.

^7^Replacement with a blank screen was done to ensure that the button press could not be used as a strategy to finish the experiment more quickly.

^8^Only participants who provided data in all conditions relevant to each particular test were included (e.g., only participants who terminated at least one encounter with each of the six agent types were included in analysis of termination frequencies). Thus, due to listwise deletion of participants with missing data, the number of observations (and consequently the degrees of freedom) vary across tests. In addition, while the proportion of encounters terminated due to being unnerved is the only rationale item central to our hypotheses, all data for participants' rationale is included (including rationale for electing to view photographs in full) for completeness of reporting.

^9^Cohen's *d*_*z*_, corrected for the within-subjects design per Morris and DeShon (2002), is reported for all significant contrasts.

^10^In testing the valley hypothesis (H1), the set of agents of *high* human similarity (40) includes both robots and people of the *atypical* and *ambiguous* designations. Note also that the set of agents of *prototypic* human similarity (10) refers only to the set of people of prototypically human appearances.

^11^We speculate that this asymmetry in perception of atypicality stems from two potential sources. First, participants may have been uncomfortable in making explicit atypicality ratings of persons with prostheses, and thus the ratings may not be representative of participants' actual perception. Second, participants exposure to persons wearing black, full-sclera contacts is likely lower than that of exposure to persons with prostheses. Thus, by definition, black eyes are likely perceived as more atypical of humans than prostheses.

^12^In testing the underlying mechanisms (M1–M2), the set of agents of *prototypic* typicality (20) includes both mechanomorphic robots and prototypic persons. Note also that the set of agents of *atypical* (20) and *ambiguous* (20) agents is inclusive of both robots and people.

^13^We note, however, that the results of the manipulation checks leave us unable to assert this implication definitively. Specifically, participants rated the set of “ambiguous” human stimuli as *more* atypical than those intended to comprise the “atypical” set (comparable to their robotic counterparts). Though we suggested that the atypicality ratings of the atypical human stimuli may have been reduced (due to participants' discomfort at making explicit ratings), without resolution of the manipulation check outcome (divergence from what was expected), we are unable to assume that the asymmetry in responding to human stimuli – that is, the more aversive responding to stimuli of ambiguous humanness – is driven by category ambiguity alone.

^14^Relative only to robots of low human similarity.
